# Quercetin ameliorates chromium toxicity through improvement in photosynthetic activity, antioxidative defense system; and suppressed oxidative stress in *Trigonella corniculata* L.

**DOI:** 10.3389/fpls.2022.956249

**Published:** 2022-11-10

**Authors:** Muhammad Ashfaq Aslam, Shakil Ahmed, Muhammad Saleem, Anis Ali Shah, Adnan Noor Shah, Mohsin Tanveer, Hayssam M. Ali, Rehab Y. Ghareeb, Mohammad E. Hasan, Jallat Khan

**Affiliations:** ^1^Institute of Botany, University of the Punjab, Lahore, Pakistan; ^2^Department of Botany, Division of Science and Technology, University of Education, Lahore, Pakistan; ^3^Department of Agricultural Engineering, Khwaja Fareed University of Engineering and Information Technology, Rahim Yar Khan, Punjab, Pakistan; ^4^Tasmanian Institute of Agriculture, University of Tasmania, Hobart, TAS, Australia; ^5^Botany and Microbiology Department, College of Science, King Saud University, Riyadh, Saudi Arabia; ^6^Plant Protection and Biomolecular Diagnosis Department, Arid Lands Cultivation Research Institute, The City of Scientific Research and Technological Applications, New Borg El Arab, Egypt; ^7^Bioinformatics Department, Genetic Engineering and Biotechnology Research Institute, University of Sadat City, Sadat City, Egypt; ^8^Department of Chemistry, Khwaja Fareed University of Engineering and Information Technology, Rahim yar Khan, Punjab, Pakistan

**Keywords:** chromium, antioxidant enzymes, growth, heavy metals, fenugreek

## Abstract

Environmental stresses, including heavy metals accumulation, have posed an immense threat to the agricultural ecosystem, leading to a reduction in the yield of crucial crops. In this study, we evaluated the role of quercetin (Qu) in the alleviation of chromium (Cr) stress in Fenugreek (*Trigonella corniculata* L.). Different levels of Qu were prepared during the experiment, i.e., 15, 25, and 40 μM. For Cr toxification in potted soil, potassium chromate (K_2_Cr_2_O_7_) was used. Cr toxification reduced growth of *T. corniculata* seedlings. Cr stress also reduced fiber, ash, moisture, carbohydrate, protein, fats, and flavonoid contents. However, seed priming with Qu improved growth and physiochemical characteristics of *T. corniculata* seedlings grown in normal and Cr-contaminated soil. Seed priming with Qu escalated intercellular CO_2_ concentration, stomatal conductance, transpiration rate, and photosynthetic rate in *T. corniculata* seedlings. Application of Qu also increased the activity of antioxidative enzymes, i.e., superoxide dismutase (SOD), catalase (CAT), ascorbate peroxidase (APX), and peroxidase (POD) in *T. corniculata* seedlings exposed to normal and Cr-contaminated soil. Application of Qu incremented the activity of SOD, POD, CAT, and APX, which were increased by 28, 22, 29, and 33%, respectively, in *T. corniculata* grown in Cr-toxic soil as compared to control treatment. Chromium stress alleviation was credited to the enhanced activity of the antioxidative defensive system in *T. corniculata* seedlings. It is proposed that Qu supplementation can be used to mitigate other abiotic stresses in plants.

## Introduction

Globally, environmental hazards have increased due to anthropogenic activities such as industrialization (Sharma et al., 2012). Excessive release of environmental toxicants like heavy metals have spoiled soil health and agricultural production (Duruibe et al., 2007; Singh et al., 2016). Plants exposed to heavy metals stresses have shown abridged growth and physiochemical characteristics (Kumar et al., 2016). Some heavy metals like Cr pose toxic effects to plants even at low concentrations (Abed el Aziz et al., 2017). Cr is a toxic metal and exists in various oxidation states, i.e., Cr (III) and Cr (VI). Cr is one of the non-essential heavy metals which reduces growth and yield of crops (Nieboer and Richardson, 1980).

Industrial units discharge a huge amount of Cr in agricultural fields (Antonkiewicz et al., 2019). Agricultural fields toxificated with elevated Cr levels disturb vegetation growth and yield (Brar et al., 2000; Coetzee et al., 2020). This noxious metal also affects various physiological processes like seed germination, root development (Li et al., 2005), photosynthetic rate, and transpiration in plants (Jobby et al., 2018). Among all the oxidation states of Cr, hexavalent Cr (Cr VI) most negatively affects the growth of numerous agronomic crops (Costa, 2003; Sharma and Dhaliwal, 2019).

In the case of developing countries, a considerable quantity of Cr enriched waste generated by industrial units is used to irrigate adjacent agricultural lands (Baran and Antonkiewicz, 2017; Turan et al., 2018). This discharge is loaded with hexavalent Cr produced by tanning, wood preservation, electroplating, pulp, and paper production. This heavy metal emancipation from industries has enhanced Cr pollutant in the environment (Shahid et al., 2017; Moreira et al., 2018). Chromium is extensively reported to cause toxicity in plants in the form of repressed plant progression, causing irreparable anatomical and ultra-structural vicissitudes, intrusive mineral nutrition, interruption in the photosynthetic and respiratory system, biomass discount, initiation of chlorosis, oxidative impairment, reduced seed count, poor sprouting, and decrease in enzymatic activity(Giri and Singh, 2017).

In Pakistan, the tanning industry is one of the foremost industries, and the second largest export producing segment. It makes up about 5% of the GDP and 7% of the overall trade earnings (Ghafoor and Zafar, 2015). The lack of appropriate wastewater disposal of the leather industry has given rise to grave ecological concerns in the country. Bashir et al. (2018) reported that at present about 800 tanneries are functional in the eastern periphery of Pakistan. Out of which 300 tannery industrial units produce 8,000–9,000 cubic meters of wastewater and about 170 tons of solid tannery waste every day in Kasur city of Pakistan. Sialkot contains nearly 252 tannery industries which discharge 1150 cubic meters of wastewater every day directly imparting noxious Cr heavy metal Batool and Hasnain (2012).

*Trigonella corniculta* L. belongs to a genus that contains about 135 species of family Leguminosae. *T. corniculata* is one of its important members and a high-yield crop which is immensely cultivated throughout the Punjab, especially in Kasur. It is famous worldwide as “Kasuri Methi” and one of the traditional and most auspicious medicinal herbs utilized for several decades due to its food eminence, nutritive prominence, and curative therapeutic properties (Jiang et al., 2017). Seeds and leaves of *T. corniculata* are rich in nutritional content and are widely used for their anti-diabetic, anti-microbial, and anti-inflammatory properties, and to treat cancer diseases (Chau and Huang, 2004). The chief nutritional ingredients of *T. corniculata* seeds contain 45–60% carbohydrates, protein (30–40%) that is rich in lysine and tryptophan, 3–4% ash, and 7.5% lipid (Kang et al., 2013; Campia et al., 2017).

Exogenous application of Qu is an effective strategy to mitigate stress in plants. Researchers have publicized that dealing with stress ameliorants lowers H_2_O_2_ by enhancing the antioxidative defensive approach (Sharma et al., 2016). Qu is not harmful, corrosive, or disturbing to plants when applied in optimum quantities. This may be a sustainable approach and an emerging drift in agronomy to uplift vegetal growth and alleviate plant stresses. Stress ameliorants encourage stress resistance, adjustment of pH, and transportation of macronutrients and micronutrients contained in soil which ultimately enhance plant growth (Singh et al.,2013; Cooke and Leishman, 2016). Seed priming with plant growth regulators is one of the methods employed for stress mitigation in plants (Jisha et al., 2013; Moulick et al., 2016, 2017). Current research was carried out to explore the potential of Qu in mitigation of Cr toxicity in *T. corniculata* exposed to Cr-stress conditions. Furthermore, the research was intended to investigate the effect of Qu on growth, antioxidant enzymes and nutritional content of *T. corniculata* grown in normal and Cr-toxic soil.

## Materials and methods

A field survey was conducted in agricultural farms being irrigated with contaminated industrial effluent. Only those farms were evaluated where crops are cultivated on an area of more than 1 acre, and were at least 4 km away from each other. Soil samples were taken by a sampling approach designed by McDonald and Martinez (1990). Cr was frequently identified in soil samples which was considered for further experimentation.

### Collection of *Trigonella corniculata* seeds

Seeds of the commonly cultivated fenugreek (*Trigonella corniculata* L.) considered as kasuri methi were procured from Ayub Agricultural Research Institute, Faisalabad. Seeds of *T. corniculata* were sterilized by keeping them in 0.5% sodium hypochlorite solution for about 2–3 min. They were then thoroughly washed with distilled water. Quercetin hydrate (Qu) was purchased from Sigma Aldrich company. Seed priming was carried out with this solution for about 8 h at room temperature. Different concentrations of quercetin (Qu) were prepared, i.e., 15, 25, and 40 μM. Following priming, seeds were properly washed with distilled water. Afterward, seeds were dried using blotting paper at room temperature.

Soil was obtained from a depth of 12″ from Botanical Garden, University of the Punjab, Lahore. Following collection, soil sterilization was carried out in autoclave at 121°C for 30 min. Soil analysis was done using the methodology employed by Ali et al. (2015). For Cr toxification, K_2_Cr_2_O_7_ was used during the study. Seeds of *T. corniculata* were primed in different concentrations of Qu, i.e., Q1 (15 μM), Q2 (25 μM), and Q3 (40 μM). Treatments designed during the experiments were Q1 (15 μM), Q2 (25 μM), and Q3 (40 μM), as well as Q1.Cr (15 μM quercetin+ Cr), Q2.Cr (25 μM quercetin+ Cr), and Q3.Cr (40 μM quercetin+ Cr). Completely randomized design (CRD) was used during the study and there were five replicates for each treatment. All treated pots were placed under natural conditions in the wire house of Botanical Garden, University of the Punjab. Thinning was carried out after 15 days. After 45 days, seedlings of *T. corniculata* were carefully uprooted and further experimentation was carried out.

### Measurement of plant growth attributes

Seedlings of *T. corniculata* were harvested after 45 days. Then, the fresh weights of root and shoot samples were recorded. Dry weight was calculated after drying the harvested samples in an oven.

### Photosynthetic pigments analysis

Fresh leaves were taken and chlorophyll extract was prepared by using acetone. Subsequently, absorbance value for chlorophyll a and chlorophyll b was calculated at 646 nm and 663 nm, respectively (Lichtenthaler and Wellburn, 1983).

### Gas-exchange characteristics and net photosynthesis rate

A portable Infra-Red Gas-Exchange Analyzer (IRGA) was used to measure net photosynthetic rate (A), intercellular CO_2_, rate of transpiration (E), stomatal conductance. Readings were taken at about 9:30 a.m. in plants facing full sunlight.

### Evaluation of proline content

The method of Bates et al. (1973) was used for the determination of proline content. For this, about 1 g of leaf sample was mixed in 3% sulfosalicylic acid and then subjected to centrifugation at 11,500 rpm. Equivalent volumes of acid ninhydrin and glacial acetic acid were supplemented in leaf samples. It was kept over a hot water bath and then ice cooled. Subsequently, 4 ml toluene was added. The superior toluene chromophore was examined at 520 nm and compared with a standard curve prepared by L-proline solution.

### Determination of hydrogen peroxide

Hydrogen peroxide (H_2_O_2_) was estimated by methodology devised by Velikova et al. (2000).

### Determination of antioxidant enzymes

Catalase (CAT) activity was measured by transformation rate analysis of H_2_O_2_ to H_2_O and O_2_ (Chance and Maehly, 1955). Reaction solution was carried out by adding a 6 ml solution of 100 mm phosphate buffer with 12 mm h and 0.2 ml extract of enzyme having 7 pH. Activity of CAT enzyme was then measured at 240 nm.

Activity of POD was measured following methodology devised by Chance and Maehly (1955). Pyrogallol phosphate buffer (6 ml), 1% H_2_O_2_ (1 ml), and enzyme extract (0.2 ml) were thoroughly mixed to prepare the solution. Then absorbance was calculated at 420 nm. Control was set by mixing all reagents excluding enzymatic extract.

Ascorbate peroxidase (APX) was analyzed using reduction in absorbance produced by preparation of ascorbic acid at 290 nm in 2 ml reaction solution composed of 100 mm phosphate buffer at 7.6 pH, 0.2 mm Na-EDTA, 24 mm H_2_O_2_, 0.5 mm ascorbic acid (Cakmak, 1994).

### Seed analysis

Analysis of *T. corniculata* seed parameters, i.e., moisture, ash, protein, fat, fiber, and flavonoid was done by methodology devised by AOAC (2012).

### Determination of moisture

Moisture content was calculated by using the oven method:


$$\%\mathrm{Moisture}=W1-{\mathrm{W2}}^{*}\;100/\mathrm{W1}$$

W1 = Weight (g) of the sample before drying.

W2 = Weight (g) of the sample after drying.

### Determination of ash

Minerals have a low volatility as compared to other food components and are not demolished by heating. After heating at 450°C-600°C, all carbon mixtures (organic) were incinerated as CO_2_. The residual portion which is inorganic in nature (minerals) was considered as ash. Empty crucibles were weighed with accuracy after being incinerated. Crucibles were then cooled at room temperature. A thoroughly mixed sample (2 g) was weighed with accuracy in crucibles and kept in a muffle furnace at 600°C. Ignition was then carried out until the appearance of light grayish colored ash after 16 h. Crucibles were put out from the furnace and kept in a desiccator until they had cooled. Weight of crucibles with ash was accurately recorded.


$$\%\mathrm{Ash}=\mathrm{Wb}-\mathrm{Wc}/{\mathrm{Wa}}^{*}\;100$$

### Determination of crude fat

Triglycerides were analyzed in foodstuff through extraction of samples (in a dehydrated and crushed state) with the help of petroleum ether in an extraction apparatus. For this, solvent was collected and remaining contents of fat were oven dried and measured (Wa). Then, thimbles were taken and measurement was carried out using 3 g samples. Buchi glass beakers were dried at 105°C for up to 30 min and then cooled. Hereafter, 35–40 ml solvent was added and beakers were set in the Soxhlet apparatus. For temperature regulation normal water was used in the heating system. In a boiling position, a knob was fixed for approximately 40 min and then rinsed for about 30 min. Solvent was attained by blocking the extraction outlet. A beaker containing fat was detached until all solvent was taken. It was then dehydrated at 105°C for half an hour, cooled, and measured (Wb).


%Fat=Wb−Wa/Weight of sample×100

### Determination of crude fiber

For determination of crude fiber, a 2 g sample was taken and transferred in an 800 ml beaker. Subsequently, 200 ml of 0.2 N H_2_SO_4_ was added. Then, the mixture was subjected to boiling for 30 min under reflux. Later, 10 ml of NaOH was added and boiled for 30 min. A filtration apparatus was used to get filtrate. Residual material was washed with the help of warm water to eliminate surplus alkali. Dehydrated crucibles with residues were heated at about 120°C for 60 min. Then, they was cooled and measured. Residues were ignited overnight in muffle furnaces.


%Crude Fiber=Wb−Wa×100/Weight of Sample

### Determination of crude protein

For determination of crude proteins, 0.5 g samples were taken and added to digestion tubes. Kjeltabs were introduced with one in every tube. Digestion tubes were kept in stands (20 tubes at a time) and kept in a digester at 420°C. Kjeltech Auto Analyzer was started up and a quantity of samples was added in the program with protein factor. Two blanks were run before sample analysis. The prepared digestion tubes were fixed one by one in the position and the safety door was shut. As NaOH was added, the solution became alkaline. It was then turned into ammonium sulfate and NH_3_ gas which then moved out into the receiving flask containing excess boric acid. Following this, NH_3_ gas was converted to NH_4_^+^. Subsequent to these previous changes, boric acid was converted to borate ion. After titration of the ammonium borate formed with standard sulfuric acid the nitrogen and protein content were calibrated.

### Statistical analysis

The acquired data was analyzed through one-way ANOVA, by using SPSS software. Duncan’s multiple range test was employed for the separation of means for significant treatment at *p* ≤ 0.05 where stated values are the means of five replications ± SE.

## Results

### Effect of quercetin on growth of *Trigonella corniculata*

Chromium stress reduced growth parameters (root and shoot length) of *T. corniculata* plants. Supplementation of Qu increased root and shoot length as compared to control treatment. Q2 treatment significantly increased growth of *T. corniculata* seedlings grown in normal and Cr-potted soil. The Cr-toxificated effect was alleviated by application of Qu as in the case of Q1.Cr, Q2.Cr and Q3.Cr. In the current study, Cr toxicity reduced root length, shoot length, root fresh weight, shoot fresh weight, root dry matter, shoot dry matter, and leaf surface area by 35, 29, 60, 36, 46, 56, 42, and 21%, respectively, as compared to C-treatment. Among all Qu treatments, Q2 significantly reduced growth in *T. corniculata* (Table 1).

**Table 1:** Effects of Qu on root length, shoot length, root fresh weight, shoot fresh weight, root dry matter, shoot dry matter, number of leaves, and Leaf area in *T. corniculata* seedlings.

Treatments	Root length (cm)	Shoot length (cm)	Root fresh weight (mg)	Shoot fresh weight (mg)	Root dry matter (mg)	Shoot dry matter (mg)	Number of leaves	Leaf area (cm^2^)
C	8.10 ± 0.10d	35.70 ± 0.10e	300.33 ± 0.58f	4.50 ± 0.10e	76.00 ± 1.00de	695.00 ± 3.00 g	21.00 ± 1.00f	230.00 ± 10.54e
Cr	5.20 ± 0.10e	25.20 ± 0.10f	119.33 ± 0.58 g	2.90 ± 0.00f	41.00 ± 1.00f	305.00 ± 2.36 h	12.00 ± 1.00 g	283.33 ± 2.89f
Q1	10.40 ± 0.10c	56.33 ± 0.51c	502.67 ± 1.53c	7.30 ± 0.10c	112.33 ± 2.08c	1076.33 ± 5.51c	43.00 ± 2.65c	251.00 ± 0.00c
Q2	13.43 ± 0.45a	80.33 ± 1.16a	613.67 ± 3.50a	10.17 ± 0.12a	157.33 ± 2.35a	1374.00 ± 3.61a	58.33 ± 0.58a	125.67 ± 5.77a
Q3	12.03 ± 0.06b	68.23 ± 1.08b	552.33 ± 2.29b	8.60 ± 0.10b	132.00 ± 1.00b	1124.00 ± 4.58b	48.00 ± 0.00b	175.00 ± 6.24b
Q1.Cr	8.13 ± 0.12d	47.30 ± 1.47d	302.00 ± 2.65f	4.83 ± 0.38e	70.67 ± 0.58e	716.00 ± 3.61f	23.33 ± 0.58ef	137.33 ± 6.35e
Q2.Cr	9.80 ± 0.10c	60.37 ± 0.55bc	424.00 ± 3.61d	6.80 ± 0.10c	109.33 ± 2.23c	977.00 ± 2.65d	32.67 ± 2.31d	230.00 ± 10.54d
Q3.Cr	8.57 ± 0.23d	54.67 ± 3.21 cd	365.00 ± 5.00d	5.70 ± 0.10d	83.33 ± 1.93d	836.67 ± 2.52d	25.00 ± 0.00e	283.33 ± 2.89e

Data exhibit Means ± SD of five replicates. Non-identical letters specify significant dissimilarity between the treatments (*p* ≤ 0.05). C = control, Cr = 100 mg^−1^ kg Cr, Q1 = 15 μM quercetin L^−1^, Q2 = 25 μM quercetin L^−1^, Q3 = 40 μM L^−1^.

### Effect of quercetin on chlorophyll and carotenoid content of *Trigonella corniculata*

Chromium exposed *T. corniculata* seedlings showed reduction in Chl a, Chl b, and carotenoid by 45, 49, and 51%, respectively. The growth rate in stress ameliorant Q2 only treatment significantly increased with reference to other treatments. The Cr-toxic effect was alleviated by Qu levels as in the case of Q1.Cr, Q2.Cr, and Q3.Cr (Table 2).

**Table 2:** Effects of Qu on Chl a, Chl b, carotenoids, photosynthetic rate, Stomatal conductance, intercellular CO_2_ concentrations, and transpiration rate in *T. corniculata* seedlings.

Treatments	Chl. a (mg g^−^ FW)	Chl. b (mg g^−^ FW)	Carotenoids (mg g^−^ FW)	Photosynthetic rate (μmol m^2^ s^−1^)	Stomatal conductance (mmol m^−2^ s^−1^)	Intercellular CO_2_ Conc. (μmol mol^−^)	Transpiration Rate (m mol H_2_O m^−2^ s^−1^)
C	0.97 ± 0.02d	0.58 ± 0.01f	3.69 ± 0.02e	40.00 ± 1.00c	1.57 ± 0.04e	491.00 ± 3.00c	1.41 ± 0.02d
Cr	0.55 ± 0.04e	0.29 ± 0.01 g	1.71 ± 0.01f	26.00 ± 1.00d	0.92 ± 0.02f	375.00 ± 5.00d	1.06 ± 0.03e
Q1	1.94 ± 0.21b	0.91 ± 0.03c	6.10 ± 0.10b	60.00 ± 1.00b	1.70 ± 0.02c	581.67 ± 3.73b	1.53 ± 0.02 cd
Q2	2.47 ± 0.03a	1.18 ± 0.06b	7.77 ± 0.02a	76.33 ± 1.53a	1.92 ± 0.02a	698.67 ± 4.50a	1.67 ± 0.01b
Q3	2.10 ± 0.20bb	1.39 ± 0.01a	6.20 ± 0.20b	63.00 ± 0.36b	1.80 ± 0.01b	614.33 ± 3.13b	1.55 ± 0.02c
Q1.Cr	1.14 ± 0.06 cd	0.65 ± 0.01ef	4.10 ± 0.10d	41.67 ± 0.58c	1.52 ± 0.02e	501.00 ± 2.00c	1.60 ± 0.00bc
Q2.Cr	1.33 ± 0.06c	0.83 ± 0.02d	5.10 ± 0.10c	58.00 ± 1.00b	1.63 ± 0.02d	591.33 ± 3.21b	2.00 ± 0.10a
Q3.Cr	1.22 ± 0.06 cd	0.69 ± 0.02e	4.30 ± 0.20d	44.67 ± 0.06c	1.54 ± 0.01e	514.67 ± 3.03c	1.70 ± 0.05b

Data exhibit Means ± SD of five replicates. Non-identical letters specify significant dissimilarity between the treatments (*p*≤ 0.05). C = control; Cr = 100 mg kg^−1^ Cr; Q1 = 15 μM quercetin L^−1^; Q2 = 25 μM quercetin L^−1^; Q3 = 40 μM L^−1^.

### Influence of quercetin on gas exchange attributes of *Trigonella corniculata*

Chromium toxificated soil interfered with photosynthetic apparatus of the *T. corniculta* and reduced photosynthetic activity, stomatal conductance, and transpiration by 41, 24, and 25%, respectively. Chromium stress reduced number of leaves and leaf surface area in *T. corniculata* plants as compared to control treatment. Application of Q2 treatment significantly increased gas exchange characteristics in *T. corniculata* seedlings grown in normal and Cr-polluted soil (Table 2).

### Influence of Qu on proline and hydrogen peroxide content of *Trigonella corniculata*

**Figure 1:**
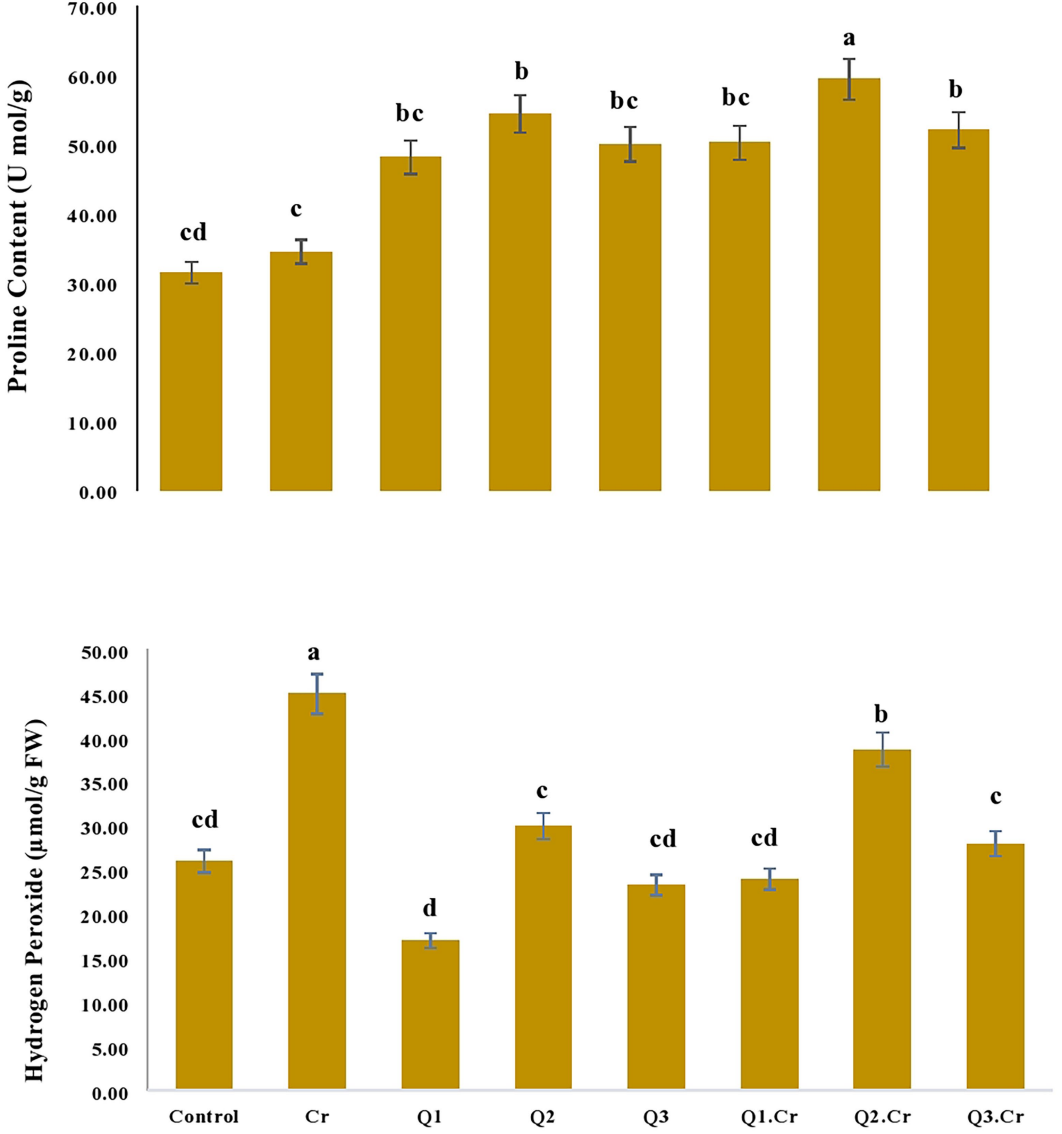
Effects of Quercetin hydrate (Q) on proline and Hydrogen peroxide contents in *T. corniculata*. Data exhibit Means ± SD of five replicates. Non-identical letters specify significant dissimilarity between the treatments (*p* ≤ 0.05). C = control, Cr = 100 mg^−1^ kg Cr, Q1 = 15 μM quercetin L^−1^, Q2 = 25 μM quercetin L^−1^, Q3 = 40 μM L^−1^.

Quantification of proline content reveals that it was improved in *Trigonella corniculta* plants with Cr-spiked soil. Application of Q2 treatment significantly increased proline content in *T. corniculata* seedlings grown in normal and Cr-toxic soil, as compared to Cr-only treatment. Likewise, Qu treatment reduced H_2_O_2_ content in *T. corniculata* seedlings grown in Cr-contaminated soil (Figure 1).

### Influence of quercetin on antioxidant enzymes of *Trigonella corniculata*

**Figure 2:**
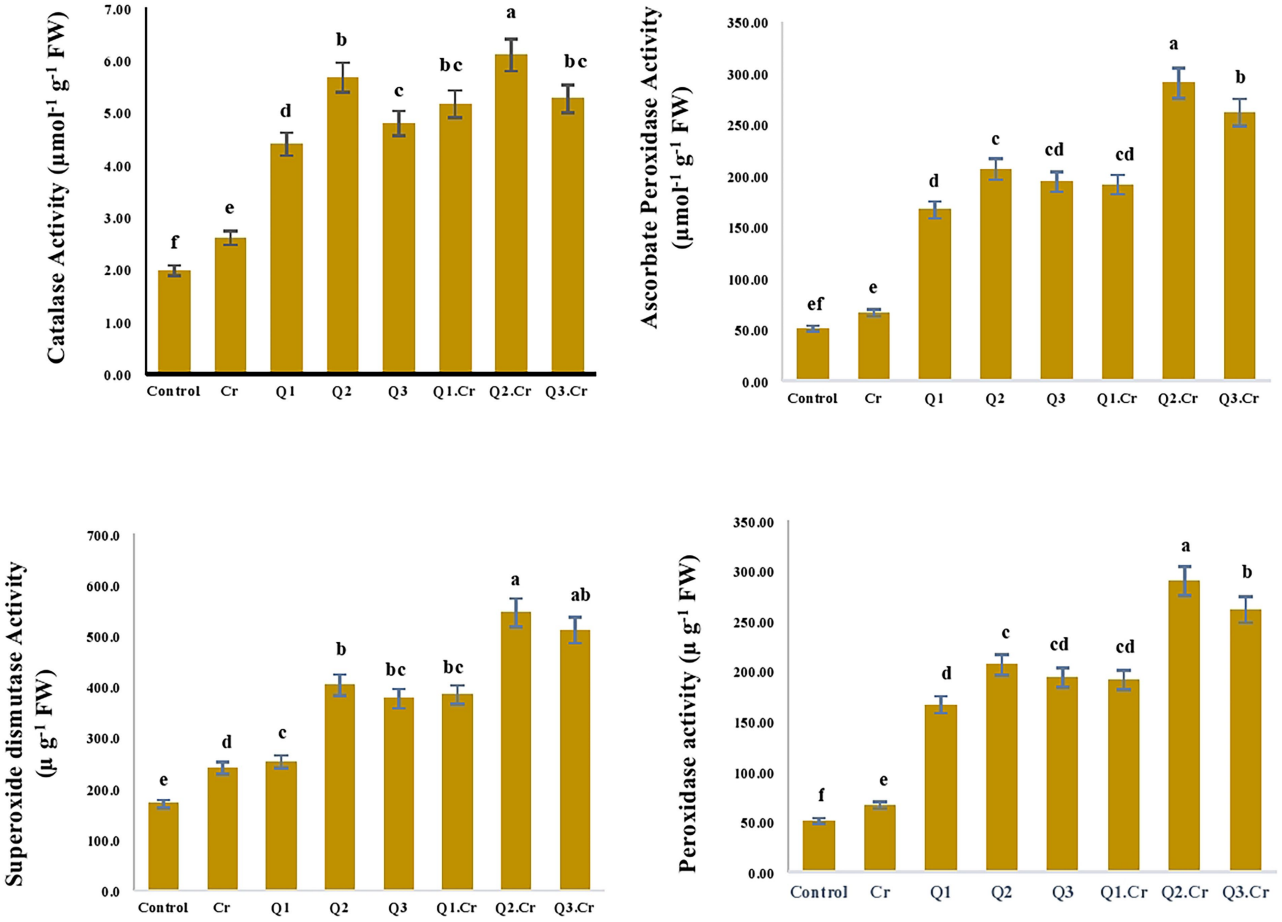
Effects of Qu on CAT, APX, SOD and POD antioxidant enzyme of seed of *T. corniculata*. Data exhibit Means ± SD of five replicates. Non-identical letters specify significant dissimilarity between the treatments (*p* ≤ 0.05). C = control, Cr = 100 mg^−1^ kg Cr, Q1 = 15 μM quercetin L^−1^, Q2 = 25 μM quercetin L^−1^, Q3 = 40 μM L^−1^.

Application of Qu decreased Cr stress in *T. corniculata* seedlings grown in Cr-contaminated soil, as compared to Cr-only treatment. As far as antioxidants enzymes are concerned, it was found that levels of SOD, POD, CAT, and APX were increased by 28, 22, 29, and 33%, respectively, in *T. corniculata* grown in Cr-toxic soil as compared to control treatment (Figure 2).

### Influence of Qu on moisture contents, ash, fiber, carbohydrate, and protein contents of *Trigonella corniculata*

**Figure 3:**
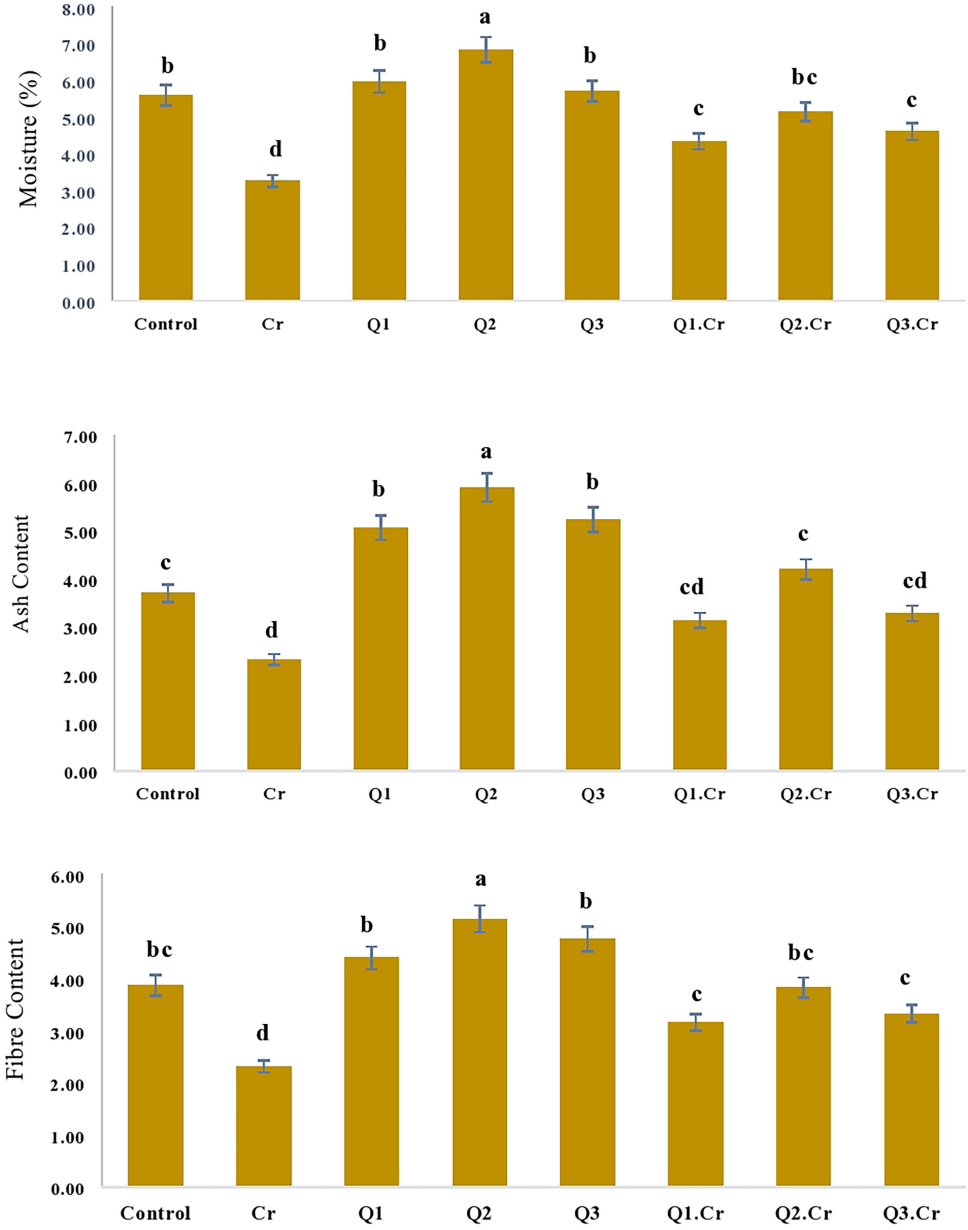
Effects of Quercetin hydrate (Q) on moisture, Ash and fiber contents of seed of *T. corniculata*. Data exhibit Means ± SD of five replicates. Non-identical letters specify significant dissimilarity between the treatments (*p* ≤ 0.05). C = control, Cr = 100 mg^−1^ kg Cr, Q1 = 15 μM quercetin L^−1^, Q2 = 25 μM quercetin L^−1^, Q3 = 40 μM L^−1^.

**Figure 4:**
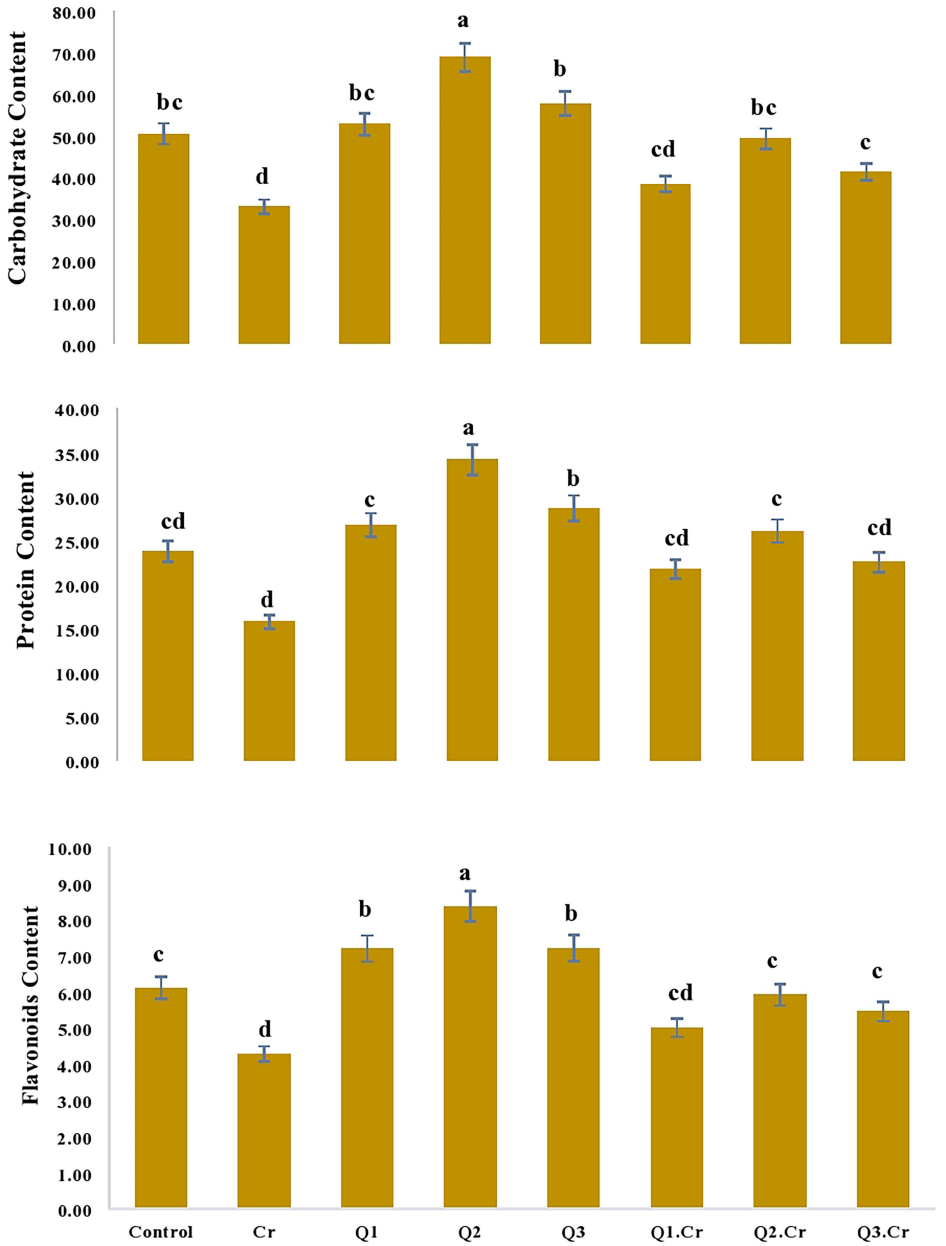
Effects of Quercetin hydrate (Q) on Carbohydrate, protein and flavonoids of seed of *T. corniculata*. Data exhibit Means ± SD of five replicates. Non-identical letters specify significant dissimilarity between the treatments (*p* ≤ 0.05). C = control, Cr = 100 mg^−1^ kg Cr, Q1 = 15 μM quercetin L^−1^, Q2 = 25 μM quercetin L^−1^, Q3 = 40 μM L^−1^.

As far as the seed contents of *T. corniculta* are concerned, Cr toxicity reduced moisture contents (42%), ash (39%), fiber (40%), carbohydrate (35%), and protein (33%) as compared to control treatment (Figures 3, 4).

## Discussion

Application of diverse phytoprotectants, either using seed priming or foliar application, has proven to be a cost-effective strategy for stress tolerance in plants as compared to traditional breeding approaches. In this section, we will explore the role of Qu in alleviation of chromium stress in *T. corniculata* seedlings.

Heavy metals are significant environmental contaminants nowadays as their increasing ecotoxicity is becoming a global public health concern (Shah et al., 2021). Seed priming is an effective strategy for stress amelioration in plants exposed to metal toxicity (Moulick et al., 2016, 2017). Quercetin is reported to protect plants against oxidative damage produced due to various abiotic stresses (Jisha et al., 2013). Previous studies have also reported that application of Qu and Qu derivatives enhanced polyphenols, antioxidant capacity of *Zea mays* seedlings (Migut et al., 2021). In our case, Qu application antioxidant capacity in *T. corniculata* seedlings exposed to Cr-stressed conditions, thereby improved growth and morphophysiological characteristics (Singh et al.,2013; Table 1).

Hydrogen peroxide is one of the stress markers in plants facing abiotic stresses. Overproduction of hydrogen peroxide (H_2_O_2_) is involved in damage to plant molecular structures (Hossain et al., 2015). Parvin et al. (2019) also reported that Qu supplementation reduced H_2_O_2_ content in tomato plants through increase in glyoxalase system and antioxidative defensive strategy. This research indicated increase in H_2_O_2_ level in *T. corniculata* seedlings exposed to Cr stress. Regulation of H_2_O_2_ content in *T. corniculata* seedlings might be involved in stress mitigation in Cr-exposed seedlings (Figure 1).

Antioxidant enzymes maintain a balance between ROS production and degradation, which is crucial for regulation of growth and morphophysiological characteristics in plants (Das and Roychoudhury, 2014). Superoxide dismutase is a crucial metalloenzyme which is involved in conversion of superoxide radical in to O_2_ and H_2_O_2_ (Río et al., 2018). Parvin et al. (2019) reported that Qu treatment mediated salt stress in tomato seedlings through enhanced activity of the antioxidative defense system. Current research revealed that Qu treatment enhanced SOD, CAT, APX, and POD activity in *T. corniculata* seedlings grown in Cr-contaminated potted soil (Figure 2).

Proline is a crucial osmoprotectant and a source to regulate nutritional content in plants exposed to abiotic stresses (Chun et al., 2018). Proline is a vital metal chelator, signaling molecule and activated antioxidative defensive approach in plants. This osmolyte brings down the level of ROS produced in plants *via* oxidative damage due to stresses (Dar et al., 2016). During the current research, regulation of proline content in *T. corniculata* seedlings exposed to Cr stress might be involved in upregulation of antioxidative enzymes and downregulation of H_2_O_2_.

**Figure 5:**
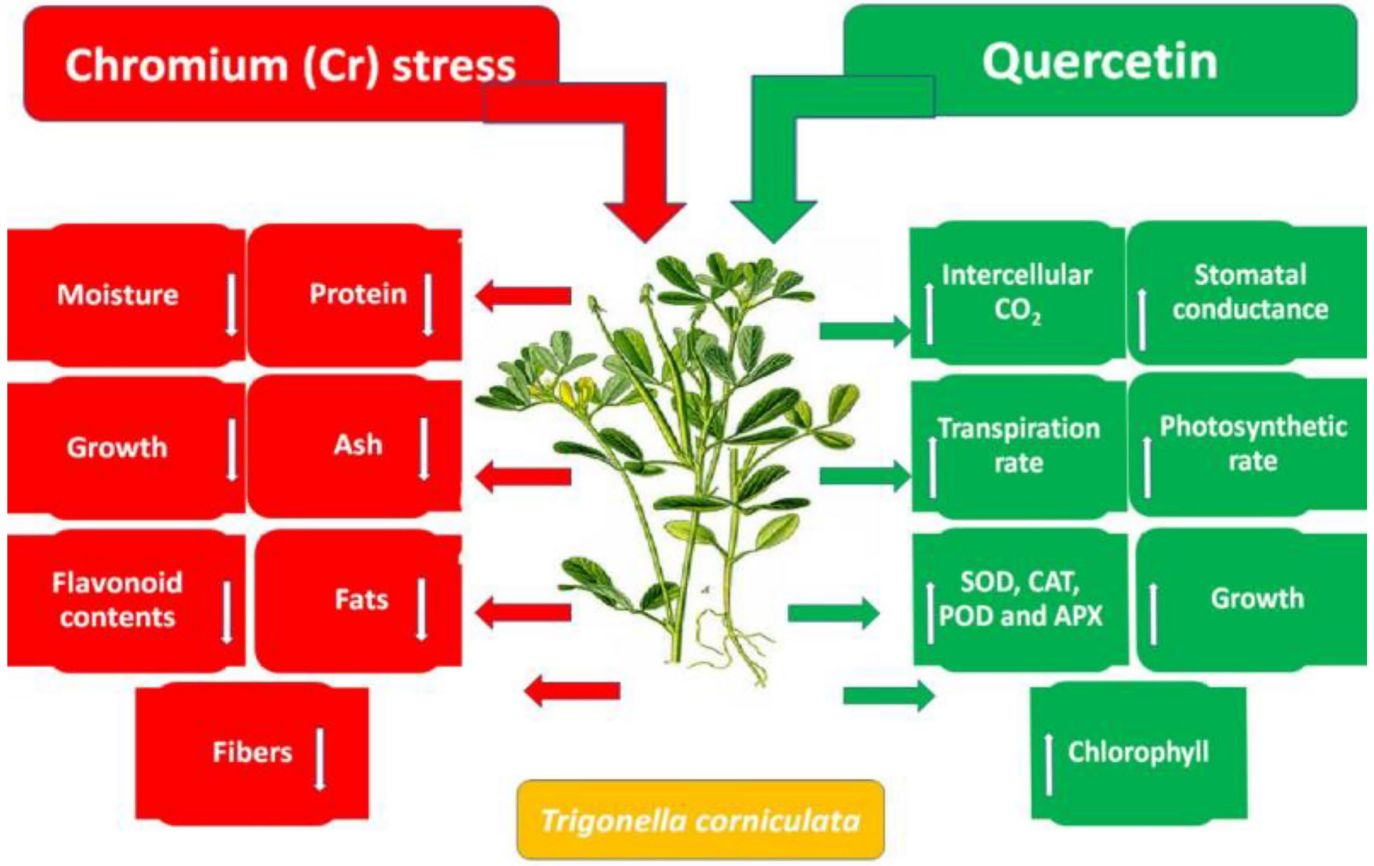
Schematic diagram showing the effect of Qu on growth and physiochemical parameters of *T. corniculata* grown in Cr-toxic potted soil.

Accumulation of flavonoids is correlated with stress tolerance in plants facing stressed conditions (Jan et al., 2021). Flavonoids are powerful antioxidants and protect plants against the damaging effects of overproduced ROS (Shah et al., 2020). Chromium stress reduced flavonoid content in *T. corniculata* seedlings grown in Cr-polluted soil. Quercetin treatment enhanced flavonoid content in *T. corniculata* seedlings grown in Cr-toxic soil. During the current study, increase in concentration of flavonoid might be involved in quenching of ROS, thereby regulating growth and physiochemical features in *T. corniculata* seedlings (Figures 4, 5). Figure 5 depicts the detailed mechanisms involved in Cr-stress alleviation in *T. corniculata* through application of Qu.

## Conclusion

The current research investigated the role of Qu in alleviation of Cr stress in *T. corniculata* seedlings. Chromium stress reduced growth and physiochemical attributes of *T. corniculata* seedlings. Contrarily, Qu treatment reversed the toxic effect of Cr on *T. corniculata* seedlings exposed to Cr-potted soil. Quercetin supplementation augmented the activity of antioxidant enzymes, as well as reduction in the quantity of some stress markers. Quercetin application also reduced H_2_O_2_ content in *T. corniculata* seedlings grown in Cr-contaminated soil. Henceforth, it is proposed that seed priming with Qu can be used in the alleviation of other abiotic stresses.

## Data availability statement

The original contributions presented in the study are included in the article/supplementary material, further inquiries can be directed to the corresponding authors.

## Author contributions

AA, Experimentation, Writing; SA and MS, supervision and research design; AAS and ANS, Review and Drafting; MT, HMA, RYG, statistical analysis, drafting, review, Statistical analysis; MEH, JK, Drafting, statistical analysis, review. All authors contributed to the article and approved the submitted version.

## Funding

This research was funded by Researchers Supporting Project number (RSP-2021/123) King Saud University, Riyadh, Saudi Arabia. 

## Conflict of interest

The authors declare that the research was conducted in the absence of any commercial or financial relationships that could be construed as a potential conflict of interest.

## Publisher’s note

All claims expressed in this article are solely those of the authors and do not necessarily represent those of their affiliated organizations, or those of the publisher, the editors and the reviewers. Any product that may be evaluated in this article, or claim that may be made by its manufacturer, is not guaranteed or endorsed by the publisher.
